# Physiological or Pathological Molecular Alterations in Brain Aging

**DOI:** 10.3390/ijms23158601

**Published:** 2022-08-02

**Authors:** Rossana Morabito, Marika Cordaro

**Affiliations:** 1 Department of Chemical, Biological, Pharmaceutical and Environmental Sciences, University of Messina, 98166 Messina, Italy; 2 Department of Biomedical, Dental and Morphological and Functional Imaging, University of Messina, 98122 Messina, Italy; cordarom@unime.it

Aging is a natural phenomenon that occurs due to a variety of loosely understood mechanisms [1]. Under physiological conditions, cells defend themselves through their sophisticated antioxidant machinery. However, when oxidants are produced in excess, or when the antioxidant defenses are ineffective, this balance can be disturbed, resulting in oxidative stress, which represents the beginning of the mechanisms that lead to aging. The brain seems to be particularly sensitive to the aging process with the appearance of neurodegenerative diseases, including Alzheimer’s disease, Parkinson’s Disease, Vascular dementia and many others. The Special Issue “Physiological or Pathological Molecular Alterations in Brain Aging “has been conceived to collect all aspects of brain studies and health promotion and prevention, such as studies on mitochondrial dysfunction, autophagy, oxidative stress and inflammation associated with brain disorders. In this regard, studies on the pharmacological, phytochemical or nutraceutical effects of compounds that are able to counteract the impact of the age in the brain have also been considered. Here, we offer an overview of the content of this Special Issue, which includes three original articles and three reviews.

Both animal model and cells have been widely used to study the effects of the aging process. Interestingly, exposure to environmental contaminants is found to be linked to an increased risk of neurological disease. In particular, atrazine (ATR) 6-chloro-N’-ethyl-N-(1-methylethyl)-1,3,5-triazine-2,4-diamine, the most commonly used broad-spectrum herbicide in agricultural crops, is released into the atmosphere following its preparation, manufacture and disposal and enters the environment, causing physiological alterations in organisms.

Several studies have demonstrated that ATR leads to induced toxicity in the brain’s neuronal circuits, in both dopaminergic and serotonergic systems, alteration of GABA, glutamate and glutamine neurotransmitters and significant behavioral alteration [2]. Specifically, in the study of Genoese and co-authors, after 28 days of daily exposure to an aerosol containing 25 mg/kg of ATR, inhalation induced a significant increase in both young and aged mice with regard to oxidative stress and lipid peroxidation in the prefrontal cortex, as well as in the hippocampus. Neurons are particularly vulnerable to oxidative damage; it is, therefore, not surprising that ROS and RNS formation, SOD, CAT, and GPx systems were significantly compromised in aged mice subjected to ATR inhalations. After exposure to ATR, the older mice developed more behavioral alterations compared to the juvenile mice, particularly anxiety, depression, spatial learning and memory function, as well as motor deficit and loss sociability. Furthermore, in the elderly mice, oxidative stress and apoptosis increased, with a reduced physiological response antioxidant system. This is the first study that considers ATR as an air contaminant that can compromise physiological aging by speeding up and interrupting the whole process [3,4].

The origin of cellular stress and neuronal loss probably stems from multiple pathways. These include (but are not limited to) bioenergetic insufficiency, neuroinflammation, and loss of proteostasis. However, cells have adapted compensatory mechanisms to overcome stress and circumvent death. One mechanism is mitophagy. Mitophagy is a form of macroautophagy, in which mitochondria are essential for synaptic function, synthesis, release and absorption of neurotransmitters. Signaling between microglia, astrocytes and neurons is modulated by the pathways of mitophagy. New data show that transcellular mitophagy occurs within the brain. It is a process by which cells release mitochondria into the extracellular space, which can be phagocytosed by the surrounding cells. Dysregulation of this process can lead to neuroinflammation and loss of proteostasis. Impairment of mitophagy is observed with aging and in many neurodegenerative diseases [5]. Reduced autophagy and mitophagy are observed in models of aging. Mitochondrial quality control emerges as a central theme in most neurodegenerative diseases, including Alzheimer’ disease (AD), Parkinson’s disease (PD), multiple sclerosis (MS), and amyotrophic lateral sclerosis (ALS). Mitophagy stimulation has shown positive effects in models of these diseases. Those designing new therapeutic strategies should focus on modulating specific mitophagy targets, while also enhancing mitochondrial function and biogenesis [6].

Despite being such long-lived cells, microglia have rarely been studied for their role in the aging process. The identification of aged microglia has proved to be difficult, due to the diversity of cell populations and the limitations of available models and the differences between human and rodent cells. The role of microglia in brain homeostasis, particularly iron storage and metabolism, may provide a key to reliable identification. Changes in microglial state are associated with changes in morphology, gene expression, and behavior. Disease-associated or activated microglia tend to have a more amoeboid form, with retracted processes and demonstrate increased phagocytosis. Dystrophic or senescent microglia show cytorrhesis and decreased phagocytosis and motility. Senescent cells are still metabolically active and able to induce changes in their environment through secreted molecules, in what has been termed the secretory-associated senescence phenotype (SASP) [7]. Therefore, SASP represents only a senescence phenotype, where the term “senescence” encompasses a wide heterogeneous range, such as microglia itself. This heterogeneity may also contribute to the differences in microglial behavior in vitro versus in vivo, which have so far represented a significant obstacle to translational research, yet have only recently been recognized [8].

Interestingly, alternative splicing and/or promoter usage occur in a great majority of mammalian genes and are key mechanisms in transcriptional regulation and generation of protein diversity. Peroxisome proliferator-activated receptor gamma coactivator 1A (PGC-1α), encoded by PPARGC1A, is a versatile transcriptional coactivator that participates in the regulation of many transcriptional programs. Functional studies in animal or cell culture models implicate PGC-1α in clinically distinct neurodegenerative diseases, including Huntington’s disease (HD), Parkinson’s disease (PD), Alzheimer’s disease (AD), and amyotrophic lateral sclerosis (ALS). Immune-mediated CNS disorders, such as multiple sclerosis, have been also linked to PGC-1α [9,10]. RNA sequencing and genetic studies in humans have also suggested that the PPARGC1A locus modulates the risk of AD, HD, PD, and ALS [7,8,9]. As some of these associations include the CNS-specific region of the PPARGC1A locus, CNS-specific isoforms and/or their control may be involved in these disorders [10].

Martínez-Iglesias and co-authors focused on the fact that AD is a complex disorder in which genomic, epigenomic, cerebrovascular, metabolic and environmental factors are potentially involved. A novel triple transgenic (3xTg)-AD mouse model was used in their study, in which the mice expressed the Swedish mutation of human amyloid precursor protein (APP), bridge integrator 1 (BIN1) and homologous photomorphogenic subunit constitutive COP9 5 (COPS5). These APP/BIN1/COPS5 3xTg-AD mice exhibited Aβ and tau pathology, a high level of anxiety and fea, severe neuropathological degeneration, and deficits in synaptic plasticity, object recognition, and learning. Epigenetics is the study of reversible heritable changes in gene expression that occur without changes to the DNA sequence, linking the genome and the environment. The accumulation of epigenetic alterations throughout one’s lifespan may lead to cerebrovascular and neurodegenerative disorders. Classic epigenetic mechanisms include DNA methylation, chromatin remodeling/histone modifications, and micro RNA (miRNA) regulation, and affect gene expression patterns. An APP/BIN1/COPS5 3xTg-AD mouse model is reliable for developing and testing novel epigenetic biomarkers or epidrugs against AD pathology [11].

The use of natural antioxidants and nutraceuticals in neurodegenerative diseases is the subject of intense research. Long-term studies with regular tree nut consumption have indicated positive outcomes for multiple health benefits. In particular, Gervasi and co-authors reviewed the beneficial effects of tree nuts, highlighting the impact on glucose modulation, body weight management, cardiovascular risk, inflammation, oxidative stress, cognitive performance, and gut microbiota. This author denoted that nut consumption was shown to significantly improve endothelial function, which is an important risk factor for cardiovascular disease (CVD). The positive effects of nut consumption on various cardiovascular disease risk factors included improvements in triglycerides, total cholesterol (TC), and lipoprotein cholesterol. Nuts, mainly almonds and pistachios, have a positive effect on the composition of the bacterial and fungal fecal microbiota. In fact, nutrients and phytochemicals can enter the colon intact and elicit their beneficial action. Almonds are rich in nutrients that promote cognitive function. In fact, numerous studies have identified an effect of almond consumption on cognitive performance. Since neurons are particularly susceptible to oxidative stress, foods rich in omega-3 fatty acids, such as walnuts, can help build and repair damaged brain cells. Phenols, polyphenols and vitamins also reduce the onset of cellular oxidative stress, inflammation and activation of the apoptotic pathway, which could be directly linked to brain aging and neurodegeneration [12].
